# Supplementary material for: Reproductive biology of long-whiskered catfish (Mystus aor) relative to temperature and precipitation from the Kaptai Lake of Bangladesh
Source: PLoS One. 2024 Nov 11;19(11):e0312835. doi: 10.1371/journal.pone.0312835 (PMC11554141; doi:10.1371/journal.pone.0312835)
Supplement: Attachment — Submitted filename: Dr. Ravindra Pawar - Authors Comments.pdf [file pone.0312835.s001.pdf]

This review concerns manuscript PONE-D-24-35998 titled "Reproductive biology of long-whiskered catfish (*Mystus aor*) relative to temperature and precipitation from the Kaptai Lake of Bangladesh." My comments follow.

Overall summary:

- The study addresses an important topic in fisheries biology and is crucial for fisheries management, conservation, and aquaculture development, especially in regions where fish populations are under pressure from overexploitation and environmental changes.
- A clear justification has been provided for the study by highlighting the economic importance of the study species *Mystus aor*, the declining fish stocks in Kaptai Lake, and the lack of recent studies on this species in the context of changing climatic conditions. The need for updated biological data to inform conservation and management strategies has been well articulated.
- Established methods have been used for analyzing reproductive biology parameters namely length-weight relationships, condition factors, gonadosomatic index, hepatosomatic index, fecundity, and histological analysis of gonads.
- Data have been collected over a full year cycle to capture seasonal variations.
- Long-term environmental data (40 years) have been analyzed to contextualize the findings and appropriate statistical analyses like ANOVA, t-tests, and correlation analyses have been used.

Specific comments:

- Sample size: Monthly about 136 fish samples were collected [Line 174]. It remains unclear how many fish were analyzed for each studied parameter. The study could benefit from a more detailed explanation of how the sample size was determined and put to use to capture seasonal and interannual variability. There is no mention of

whether the sample size was sufficient to detect meaningful differences in the parameters measured. This information would help in assessing the robustness of the findings.

- Abiotic parameters: While precipitation and temperature data are analyzed, other potentially important factors, for example, dissolved oxygen, pH, water flow rates, etc. Have not been considered.
- Correlation between environmental factors (e.g., precipitation) and reproductive parameters (spawning) would benefit if presented in the form of a statistical analysis.
- Given the multiple parameters measured, a multivariate analysis might have provided additional insights into how different factors interact to influence reproductive biology.
- Confounding factors: While the findings mention overfishing and pollution as threats, very limited discussion is provided on how these factors might have influenced their results.
- The discussion would benefit if a broader comparison with similar studies or species is included.
- References:
    1. Missing in-text citation: [22] Talwar and Jhingran [Line 623].
    2. Missing in 'References': [27] Ranganathan and Radha [Line 438].
- Spellings:
    1. 'Bagridae' NOT 'Bagaridae' [Line 143].
    2. 'recorded' NOT 'recored' [Line 438].
