# Supplementary material for: Reproductive biology of long-whiskered catfish (Mystus aor) relative to temperature and precipitation from the Kaptai Lake of Bangladesh
Source: PLoS One. 2024 Nov 11;19(11):e0312835. doi: 10.1371/journal.pone.0312835 (PMC11554141; doi:10.1371/journal.pone.0312835)
Supplement: Attachment — Submitted filename: Response_to_reviewer.docx [file pone.0312835.s002.docx]

Editor-in-Chief  
PLOS ONE

17^th^ September, 2024

Dear Editor-in-Chief

We have revised our manuscript titled “**Reproductive biology of long-whiskered catfish (*Mystus aor*) relative to temperature and precipitation from the Kaptai Lake of Bangladesh**” (PONE-D-24-35998), according to the editors’ and the reviewers’ suggestions. We appreciate the comments and have accepted most of the suggestions, as detailed below with line numbers of the revised manuscript in the responses.

We have revised the manuscript which improved the insight of the study. The details of arrangement and modifications are described in the responses to the reviewers.

We do hope that the manuscript is now suitable for publication.

Yours sincerely,

Md. Moudud Islam

------------------------------------

**Response to the suggestion and comments of the reviewer**

When submitting your revision, we need you to address these additional requirements.

1. Please ensure that your manuscript meets PLOS ONE's style requirements, including those for file naming. The PLOS ONE style templates

**Response: Sorry for our unintentional mistake. We formatted the whole manuscript as per PLOS ONE style templates.**

2. We note that the grant information you provided in the ‘Funding Information’ and ‘Financial Disclosure’ sections do not match.

When you resubmit, please ensure that you provide the correct grant numbers for the awards you received for your study in the ‘Funding Information’ section.

**Response: Sorry for our unintentional mistake. ‘Funding Information’ and ‘Financial Disclosure’ are now same**

3. Thank you for stating the following financial disclosure:

“CVASU-University Grant Commission”

Please state what role the funders took in the study. If the funders had no role, please state: "The funders had no role in study design, data collection and analysis, decision to publish, or preparation of the manuscript." If this statement is not correct you must amend it as needed.

Please include this amended Role of Funder statement in your cover letter; we will change the online submission form on your behalf.

**Response: Financial disclosure has been added in the cover letter.**

4. Thank you for stating the following in the Acknowledgments Section of your manuscript:

“The authors wish to express their gratitude to the UGC-CVASU of Bangladesh FY 2020– 21 for funding this research. The authors also express sincere thanks to the fishers and fish sellers who helped to collect samples and all of the laboratory personnel of the Fish Biology and Biotechnology Department, CVASU, for their kind support.”

We note that you have provided funding information that is not currently declared in your Funding Statement. However, funding information should not appear in the Acknowledgments section or other areas of your manuscript. We will only publish funding information present in the Funding Statement section of the online submission form.

Please remove any funding-related text from the manuscript and let us know how you would like to update your Funding Statement. Currently, your Funding Statement reads as follows:

“CVASU-University Grant Commission”

Please include your amended statements within your cover letter; we will change the online submission form on your behalf.

**Response: We understand the guideline of the journal. Funding information is removed from the acknowledgment section of the manuscript.**

5. Please provide a complete Data Availability Statement in the submission form, ensuring you include all necessary access information or a reason for why you are unable to make your data freely accessible. If your research concerns only data provided within your submission, please write "All data are in the manuscript and/or supporting information files" as your Data Availability Statement.

**Response: Data related to this study obtained from the year-round sampling and the obtained data is not suitable for submission for any repository. However, data can be available upon request to the corresponding author.**

6. Please include captions for your Supporting Information files at the end of your manuscript, and update any in-text citations to match accordingly. Please see our Supporting Information guidelines for more information: <http://journals.plos.org/plosone/s/supporting-information>.

**Response: There is no supporting information files related to the manuscript.**

7. Please review your reference list to ensure that it is complete and correct. If you have cited papers that have been retracted, please include the rationale for doing so in the manuscript text, or remove these references and replace them with relevant current references. Any changes to the reference list should be mentioned in the rebuttal letter that accompanies your revised manuscript. If you need to cite a retracted article, indicate the article’s retracted status in the References list and also include a citation and full reference for the retraction notice.

**Response: Thank you for your comments. There was no reference which has been retracted from the manuscript. Reference list has been checked and completed as per journal style.**

Reviewers' comments:

Reviewer's Responses to Questions

**Comments to the Author**

1. Is the manuscript technically sound, and do the data support the conclusions?

The manuscript must describe a technically sound piece of scientific research with data that supports the conclusions. Experiments must have been conducted rigorously, with appropriate controls, replication, and sample sizes. The conclusions must be drawn appropriately based on the data presented.

Reviewer #1: Yes

2. Has the statistical analysis been performed appropriately and rigorously?

Reviewer #1: Yes

3. Have the authors made all data underlying the findings in their manuscript fully available?

The [PLOS Data policy](http://c05y1x9s.r.us-east-2.awstrack.me/L0/http:%2F%2Fwww.plosone.org%2Fstatic%2Fpolicies.action%23sharing/1/010f0191e0568c8c-9a3ae209-f84e-45c9-86cb-a0c8acdac916-000000/vm85KCD7NnTIYSJzQfGo63DiQO0=176) requires authors to make all data underlying the findings described in their manuscript fully available without restriction, with rare exception (please refer to the Data Availability Statement in the manuscript PDF file). The data should be provided as part of the manuscript or its supporting information, or deposited to a public repository. For example, in addition to summary statistics, the data points behind means, medians and variance measures should be available. If there are restrictions on publicly sharing data—e.g. participant privacy or use of data from a third party—those must be specified.

Reviewer #1: Yes

4. Is the manuscript presented in an intelligible fashion and written in standard English?

PLOS ONE does not copyedit accepted manuscripts, so the language in submitted articles must be clear, correct, and unambiguous. Any typographical or grammatical errors should be corrected at revision, so please note any specific errors here.

Reviewer #1: Yes

**Response: Thanks to the reviewer for his thoughtful consideration regarding the importance of our manuscript. We appreciate reviewer’s concern.**

5. Review Comments to the Author

Please use the space provided to explain your answers to the questions above. You may also include additional comments for the author, including concerns about dual publication, research ethics, or publication ethics. (Please upload your review as an attachment if it exceeds 20,000 characters)

Reviewer #1: Detailed observations on Q. 1 to 4 above have been included in the attached document. However, here I am pasting them again.

This review concerns manuscript PONE-D-24-35998 titled "Reproductive biology of long-whiskered catfish (Mystus aor) relative to temperature and precipitation from the Kaptai Lake of Bangladesh." My comments follow.

Overall summary:

- The study addresses an important topic in fisheries biology and is crucial for fisheries management, conservation, and aquaculture development, especially in regions where fish populations are under pressure from overexploitation and environmental changes.
- A clear justification has been provided for the study by highlighting the economic importance of the study species Mystus aor, the declining fish stocks in Kaptai Lake, and the lack of recent studies on this species in the context of changing climatic conditions. The need for updated biological data to inform conservation and management strategies has been well articulated.
- Established methods have been used for analyzing reproductive biology parameters namely length-weight relationships, condition factors, gonadosomatic index, hepatosomatic index, fecundity, and histological analysis of gonads.
- Data have been collected over a full year cycle to capture seasonal variations.
- Long-term environmental data (40 years) have been analyzed to contextualize the findings and appropriate statistical analyses like ANOVA, t-tests, and correlation analyses have been used.
- **Response: We appreciate reviewer’s positive thought regarding our manuscript.**

Specific comments:

- Sample size: Monthly about 136 fish samples were collected [Line 174]. It remains unclear how many fish were analyzed for each studied parameter. The study could benefit from a more detailed explanation of how the sample size was determined and put to use to capture seasonal and interannual variability. There is no mention of whether the sample size was sufficient to detect meaningful differences in the parameters measured. This information would help in assessing the robustness of the findings.

**Response: We appreciate reviewer’s comment. In this study, we have collected 10−17 fish of both sexes monthly. All the collected fish (both male and female) were taken into considerations for studying the reproductive and seasonal variation in the maturation pattern. Line 170−171 and 174−176.**

- Abiotic parameters: While precipitation and temperature data are analyzed, other potentially important factors, for example, dissolved oxygen, pH, water flow rates, etc. Have not been considered.

**Response: We appreciate reviewer’s comment. We did not collect and analyzed the abiotic parameters for this study since there are many published data about the abiotic parameters in the Kaptai lake and found optimum level in the Kaptai lake. We added the information in line number 544.**

- Correlation between environmental factors (e.g., precipitation) and reproductive parameters (spawning) would benefit if presented in the form of a statistical analysis.

**Response: We appreciate reviewer comments. We have analyzed the correlation between GSI value with rainfall and temperature and added in the results section and new figures have been added as 9C and 9D. Line number 364−371.**

- Given the multiple parameters measured, a multivariate analysis might have provided additional insights into how different factors interact to influence reproductive biology.

**Response: We appreciate reviewer’s comment. Since multivariate analysis like PCA or DFA is widely used for determining the interactions for a given characteristic. In this study, we first determine the reproductive biology of *M. aor* by observing the different parameters which is just some indicator (not a determining factor for spawning) to assess the reproductive seasonality. In addition, the histological analysis of gonad, is the key indicator for assessing the reproductive seasonality and spawning pattern which could be difficult to mathematically expressed to set for multivariate analysis. Moreover, we have just studied temperature and rainfall as a factor which could influence reproductive biology, that we have already explain in the results and discussion section. If we have multiple other factors that positively or negatively influence the reproduction, we might have done multivariate analysis. Currently, we are working on other biotic and abiotic factors, reproductive biology of some other fish species, fishing pressure, and pollution in the Kaptai lake and we have plan to analyze the collected data through multivariate analysis in our next manuscript.**

- Confounding factors: While the findings mention overfishing and pollution as threats, very limited discussion is provided on how these factors might have influenced their results.

**Response: We appreciate reviewer’s comment. Details of confounding factors especially pollutants and overfishing has already been discussed in line number 527−533. Additional supporting information is added in line number 533−536.**

- The discussion would benefit if a broader comparison with similar studies or species is included.

**Response: We appreciate reviewer’s comment. To date very few studies have been performed on the reproductive biology on the studied species and we have already cited in the manuscript with details comparison. In addition, relationship between environmental factors and spawning have been described only a few species. Taking considerations of the reviewer’s comment, we have added a broader comparison in the discussion section line 517−526.**

- References:
  Missing in-text citation: [22] Talwar and Jhingran [Line 623].

**Response: Talwar and Jhingran, [22] was already cited in the text in line number 141 and 149.**

- Missing in ‘References’: [27] Ranganathan and Radha [Line 438].

**Response: Sorry for our mistake. Reference is added in the reference section [46]**

- Spellings:
  1.‘Bagridae’ NOT ‘Bagaridae’ [Line 143].
  2.‘recorded’ NOT ‘recored’ [Line 438].

**Response: Sorry for the typological mistake. We revised the manuscript.**
